# Toxicological Assessment of Biodegradable Poli-ε-Caprolactone Polymer Composite Materials Containing Hydroxyapatite, Bioglass, and Chitosan as Potential Biomaterials for Bone Regeneration Scaffolds

**DOI:** 10.3390/biomedicines12091949

**Published:** 2024-08-26

**Authors:** Aleksandra Skubis-Sikora, Andrzej Hudecki, Bartosz Sikora, Patrycja Wieczorek, Mateusz Hermyt, Marek Hreczka, Wirginia Likus, Jarosław Markowski, Krzysztof Siemianowicz, Aleksandra Kolano-Burian, Piotr Czekaj

**Affiliations:** 1 Department of Cytophysiology, Chair of Histology and Embryology, Faculty of Medical Sciences in Katowice, Medical University of Silesia in Katowice, 40-055 Katowice, Poland; askubis@sum.edu.pl (A.S.-S.); bsikora@sum.edu.pl (B.S.); pszmytkowska@sum.edu.pl (P.W.); mhermyt@sum.edu.pl (M.H.); 2 Łukasiewicz Research Network-Institute of Non-Ferrous Metals, 44-121 Gliwice, Poland; andrzej.hudecki@imn.lukasiewicz.gov.pl (A.H.); marek.hreczka@imn.lukasiewicz.gov.pl (M.H.); aleksandra.kolano-burian@imn.lukasiewicz.gov.pl (A.K.-B.); 3 Department of Anatomy, Faculty of Health Sciences in Katowice, Medical University of Silesia in Katowice, 40-055 Katowice, Poland; wirginia.likus@gmail.com; 4 Department of Laryngology, Faculty of Medical Sciences in Katowice, Medical University of Silesia in Katowice, 40-055 Katowice, Poland; jmarkowski@sum.edu.pl; 5 Department of Biochemistry, Faculty of Medical Sciences in Katowice, Medical University of Silesia in Katowice, 40-055 Katowice, Poland; ksiemian@gmail.com

**Keywords:** poli-ε-caprolactone, hydroxyapatite, chitosan, bioglass, toxicity, composite material scaffolds, bone tissue regeneration, stem cells

## Abstract

Polycaprolactone (PCL) is a biodegradable polyester that might be used in tissue engineering to obtain scaffolds for bone reconstruction using 3D-printing technologies. New material compositions based on PCL, with improved physicochemical properties and excellent biocompatibility, would improve its applicability in bone regeneration. The aim of this study was to assess the potential toxic effects of PCL-based composite materials containing 5% hydroxyapatite (PCL/SHAP), 5% bioglass (PCL/BIO), or 5% chitosan (PCL/CH) on MG-63 human fibroblast-like cells in vitro. Material tests were carried out using X-ray diffraction, differential thermal analysis/thermal gravimetry, BET specific surface analysis, and scanning electron microscopy. The effect of the biomaterials on the MG-63 cells was then assessed based on toxicity tests using indirect and direct contact methods. The analysis showed that the tested biomaterials did not significantly affect cell morphology, viability, proliferation, or migration. We concluded that biodegradable PCL-based scaffolds may be suitable for tissue scaffold production, and the addition of bioglass improves the growth of cultured cells.

## 1. Introduction

Currently, the gold standard in bone reconstruction procedures is autologous bone transplantation [1]. It is associated with specific osteoconductive, osteogenic, and osteoinductive properties of bones [2,3]. Osteoconduction is the ability of cells to grow on the graft surface. On the other hand, osteoinduction is a process promoting osteogenesis involving the differentiation of immature cells into preosteoblasts [4]. Moreover, autologous grafts recreate the mechanical properties of the replaced bone and are not immunogenic [2].

However, the growing demand for modern treatment methods in bone engineering is intensifying the development of biomaterial-based grafts. Tissue engineering focuses on the replacement of damaged tissues with cells seeded onto biomaterials capable of restoring their original form and properties. Successfully bioengineered tissue is composed of a biocompatible material that serves as a scaffold carrying and supporting cells driven to restore the natural-like cell network constituting the functioning tissue [5]. Scaffolds should have a specific structure aimed at replacing damaged tissue and promoting its regeneration. They must meet medical, biological, and mechanical criteria. For tissue reconstruction, scaffolds must be biocompatible, meaning they should positively interact with the surrounding cells and mimic the extracellular environment [5,6]. Recently, a number of synthetic biomaterials have been investigated, such as polylactic acid (PLA) [7,8], polycaprolactone (PCL) [9,10,11], poly(L-lactide-co-caprolactone) (PLCL) [12], poly(lactic-co-glycolic acid) (PLGA) [13,14], and poly(glycolic acid) (PGA) [15]. On the other hand, there are various natural materials promoting osteogenesis, such as tricalcium phosphate (β-TCP) [16], type I collagen [17], hydroxyapatite [18], chitosan [19], and alginate [20]. Combining natural materials with biopolymers could improve their properties that are desirable in bone reconstruction.

Effective personalized scaffold production can be achieved by 3D-printing methods, where biomaterials composed of titanium alloys or ceramic-based materials are widely used [21,22]. However, the conditions that prevail in the human body include a mostly constant temperature of 36.6 °C, an aqueous environment, the presence of enzymes, proteins, and cellular metabolites, and physical forces acting on the material. Taken together, they can, over time, cause stresses that favor the formation of microcracks in the structure. Under natural conditions, the resulting microcracks undergo a physiological regenerative process. Replacing natural tissue with a ceramic or metal biomaterial may lead to larger fractures requiring re-implantation. Therefore, solutions are being sought that (i) can temporarily take over the function of natural tissue, (ii) create an optimal environment for tissue growth, and (iii) degrade gradually while their products are incorporated into natural metabolic pathways, ultimately (iv) allowing for full tissue regeneration. Such attributes are offered by biodegradable polymeric materials [23] and composite materials, which, once implanted in the recipient’s body, are expected to degrade into easily metabolized products.

Ongoing research on polymeric materials includes PCL [24], PLA [25], and polyethylene oxide (PEO), as well as their combinations with chitosan [26], collagen, and elastin. Biodegradable polymers are of main interest in 3D printing, including fused deposition modeling (FDM). FDM printing feeds a thermoplastic filament into a nozzle, where it is melted and precisely distributed to build a given 3D model. PCL is a biodegradable polyester with a low melting point and a wide range of applications. It can be used in FDM printing as a filament. The advantages of PCL include ease of melting, malleability, and flexibility, allowing complex shapes to be formed [27]. Moreover, PCL is used in combination with polymers such as polyethylene glycol (PEG) for drug delivery [28]. Due to its long biodegradation time, this polymer will take much longer to release the drug than, for example, the equally widely used PLA. Other applications of PCL include packaging, where it is an alternative to plastics, adhesives, and textiles that are intended to imitate the physical properties of skin [29]. However, PCL has several disadvantages, such as poor mechanical properties and a tendency to swell.

Materials such as PCL, bioglass (BIO), hydroxyapatite (SHAP), and chitosan (CH) are commonly applied in medicine and serve roles in filling tissue defects, stimulating osteogenesis in bone tissue, or as components in wound dressing materials. One of the preliminary steps of a new artificial tissue construction attempt is toxicological examination of the biomaterial in terms of its potential interactions with cells in the context of their viability, proliferation rate, and functional activity. To test biomaterial biocompatibility, we used the recommended and standardized MG-63 (CRL-1427) osteosarcoma cell line, which is suitable for potential future application in bone regeneration studies. Immortalized MG-63 cells are routinely used in in vitro studies due to the relatively quick cell recovery and stability of their phenotype [30,31]. Cytophysiologically, they correspond to natural osteoblasts, although they lack ALP activity and are unable to mineralize the extracellular matrix. MG-63 cells express osteoblastic markers and have a high proliferation rate.

The aim of this study was to assess the effects of novel PCL-based composite biomaterials combined with hydroxyapatite, bioglass, and chitosan on osteoblast-like cells. We determined not only the feasibility of producing composite materials using the mentioned polymer and additives but also whether the materials would exhibit any cytotoxic effects on cells after being exposed to high temperatures.

This study specifically addresses the highly problematic aspect of searching for personalized tissue substitutes in the area of surgically removed bone tissue, where, to date, the patient’s own tissue from another part of the body is most commonly used. For this reason, we focused on subjecting the obtained materials to high temperatures to produce a filament that can potentially be used in FDM technology and then to low temperatures (through cryogenic milling) to obtain a powder that could be used in the future as a material for SLS technology, which relies on powders and allows the creation of much more complex shapes.

## 2. Materials and Methods

Pellets of PCL and its additives, hydroxyapatite, bioglass, and chitosan, were mixed mechanically and melted. The obtained composites were pulverized using a cryogenic grinder. Finally, the biomaterials were analyzed with X-ray diffraction (XRD), differential thermal analysis/thermal gravimetry (DTA/TG), Brunauer–Emmett–Teller (BET) specific surface analysis, and scanning electron microscopy (SEM). After that, the samples underwent radiation sterilization for toxicological analysis.

We investigated the influence of the biomaterial samples on MG-63 cells in vitro, namely, changes in cell morphology, proliferation, and viability; cell cycle activity; and adhesion to composite materials. Biomaterial–cell interactions were analyzed in two ways: by testing the sample extracts (elution test) and by direct contact.

### 2.1. Biomaterials

#### 2.1.1. Materials

All the biomaterials tested in this study were provided by the Łukasiewicz Research Network—Institute of Non-Ferrous Metals in Gliwice, Poland, and obtained according to the standard operating procedures and know-how of the institute. The following synthetic polymeric materials were used to produce PCL samples: single-component PCL, two-component hydroxyapatite-containing PCL/SHAP, two-component bioglass-containing PCL/BIO, and two-component chitosan-containing PCL/CH. This study used PCL with an average Mn = 80,000 (Sigma Aldrich, Saint Louis, MO, USA), chitosan of molecular weight = 100,000–300,000 (Thermo Fischer Scientific, Waltham, MA, USA), hydroxyapatite nanoparticles less than 40 nm in diameter with a purity of 98.5% (SkySpring Nanomaterials Inc., Houston, TX, USA), and bioactive glass powder less than 10 μm in diameter with a purity of 98% (Sigma Aldrich, Saint Louis, MO, USA). The composition of the bioglass provided by the manufacturer was CaO:SiO_2_:P_2_O_5_.

#### 2.1.2. Production of PCL Composite Materials

To develop the PCL/SHAP, PCL/BIO, and PCL/CH composite materials, the individual components, namely, the polymer PCL and the additives—hydroxyapatite, bioactive glass, or chitosan—were first weighed. The ratio between the PCL and each of the used components was 95:5. The initial mixtures were then placed in a 3DEVO extruder, where mechanical mixing of the components and melting at 200 °C took place to obtain a filament with a thickness of 1.75 μm. The process of combining the components involved subjecting them to an extruder in four heating zones: the first, where the material is preheated, at a temperature of 150–160 °C; the second, where the material is melted and individual granules fused together at a temperature range of 170–200 °C; the third, where the material is transported in liquid form to the fourth zone at a temperature of 190 °C; and the fourth, where the material is pressed through a nozzle at a temperature of 150–160 °C. The heating time for all the zones ranged from 30 to 90 min. After passing through the nozzle, the formed strand was cooled and directed to the cryogenic grinding process, where it was converted into a powder form.

#### 2.1.3. Preparation of PCL Powder with Added Hydroxyapatite, Bioglass, and Chitosan

The resulting composite material was placed in a 50 mL polycarbonate container closed on both sides with steel lids. Along with the composite material, a steel mandrel was placed in the container, which was set in motion between the metal lids to grind the sample. The samples thus prepared were placed in special molds and then immersed in liquid nitrogen. After the immersion process, the samples were subjected to freezing for 30 min, then 20 cycles of sample grinding were set, each of which comprised (I) an actual grinding time of 1 min and (II) an actual doming time of 2 min.

#### 2.1.4. X-ray Diffraction Studies

The phase structure of the materials was determined by wide-angle X-ray diffraction using a Rigaku MiniFlex 600 diffractometer (Rigaku, Tokyo, Japan) equipped with a copper CuKα radiation source (λ = 0.1542 nm), a Kβ Ni filter, and an ultrafast silicon D/teX detector. The tests were carried out at room temperature, in the 2θ 3–90° angle range, with a measurement speed of 1.5°/min and a measurement step of 0.02°.

The technique involves recording of the diffraction images of the X-rays produced by the subtle interactions of this radiation, with the electron clouds of the atoms constituting the analyzed material. Based on the diffraction registrations of the X-rays passing through the material at various angles, using Bragg’s law, it is possible to determine the crystalline phases in the material (or observe their absence in the case of an amorphous material). Bragg’s law is defined as follows: nλ = 2dsinθ, where n is the order of deflection, an integer but not large enough due to the fact that sin θ < 1; λ is the wavelength of the X-ray radiation, such that λ ≤ 2 d; d is the distance between the planes on which scattering occurs; and θ is the angle of incidence, defined as the angle between the beam of primary rays and the plane of the crystal.

#### 2.1.5. Calorimetric Studies

Calorimetric DTA/TG (differential thermal analysis/thermal gravimetry) tests were used to record, in a controlled manner, the temperature difference between the test substance and the reference substance with respect to time or temperature as two samples under identical conditions in a heated or cooled environment. During the test, it is possible to measure the change in mass (caused, for example, by evaporation, degradation, or the formation of an oxide layer on the material) relative to the reference sample. The heat flow (DTA) and mass change (TG) were measured using an STA Netzsch F3 Jupiter thermal analyzer (Selb, Germany) in an argon atmosphere. The heating rate was 10 K/min ranging from room temperature (25 °C) to 600 °C. The measurements were performed using Al_2_O_3_ crucibles. The characteristic temperatures and mass changes were read using the accompanying software.

#### 2.1.6. Surface Area Studies

The specific surface area was determined using a Gemini VII 2390t specific surface area analyzer (Micrometrics, Ottawa, ON, Canada). The measurement principle is based on measuring the adsorption of gas (nitrogen) on the adsorbate surface. Using this apparatus, the following aspects were measured: multi-point Brunauer–Emmett–Teller (BET) specific surface, single point BET specific surface, and Langmuir specific surface. The specific surface area is the sum of the external and internal surfaces of all the pores, expressed in m^2^ per unit mass in g of the material under investigation. By determining the specific surface area of a solid, it is possible to confirm the degree of surface development, which determines the course of physicochemical processes of the investigated material with the surrounding environment. The measuring range of the apparatus was as follows: specific surface from 0.01 m^2^/g and total surface from 0.1 m^2^ to 300 m^2^. To remove impurities and moisture, the samples were dried at a temperature of 35 °C for 2 h. The degassing of the sample was carried out under a protective nitrogen gas atmosphere. The measurement was performed in the P/P0 range from 0.05 to 0.3.

#### 2.1.7. Scanning Electron Microscopy of Biomaterials

The structure of the composite materials was examined with a ZEISS SUPRA 25 scanning electron microscope (SEM; Jena, Germany) using an accelerating voltage of 3–25 kV. The samples were sputtered with gold and then placed in the working chamber of the SEM microscope, where analysis was carried out at magnifications of 5000–10,000×. The fiber diameter and its spatial distribution were investigated using Digital Micrograph 365 software (Gatan Microscopy Suite Software, Gatan, Inc., Pleasanton, CA, USA).

#### 2.1.8. Radiation Sterilization

The composite materials obtained after cryogenic grinding were used to prepare weighted amounts of powders containing hydroxyapatite, bioglass, and chitosan, which were then used to prepare boiled lozenges with a diameter of 1 cm and a thickness of 1 mm. The resulting lozenges were packed in radiation sterilization sleeves and sent for sterilization. Sterilization was carried out at the Center for Radiation Research and Technology at the Institute of Nuclear Chemistry and Technology. A dose of 15 kGy was used for sterilization (the detailed sterilization conditions are described in Supplementary Materials, Table S1).

### 2.2. Toxicological Examination

The influence of biomaterial extracts on MG-63 cells (CRL-1427, ATCC, Manassas, VA, USA) in vitro, namely, cell morphology, proliferation, and viability, as well as cell cycle activity, were assessed by elution tests. These effects were evaluated using MTT, XTT, scratch assays, and flow cytometry. The studied groups were compared to MG-63 cells cultured in standard medium, which served as the positive control. The negative control was cells treated with standard culture medium with the addition of 1% Triton X-100.

For the direct contact assay, the cells were examined with the use of fluorescein diacetate and propidium iodide, allowing visualization of live/dead cells among the cells seeded onto the tested biomaterial samples. The adhesion of these cells to composite materials was examined by SEM.

#### 2.2.1. Preparation of Extracts for Elution Test

To prepare the extract, the biomaterials were incubated with cell culture medium at a volume of 1 mL per 0.1 g of material at 37 °C for 24 h with shaking (Enviro-Genie, Scientific Industries, Inc., Bohemia, NY, USA). The medium was then filtered and mixed with a new cell culture medium in a 1:1 ratio. The extract kept in this way was ready for cell culture.

#### 2.2.2. Cell Culture

MG-63 (CRL-1427, ATCC) human, adherent, fibroblast-like cells derived from osteosarcoma were routinely maintained in DMEM medium (Dulbecco’s Modified Eagle Medium, Gibco, Thermo Fisher Scientific, Waltham, MA, USA) supplemented with fetal bovine serum (FBS, EuroClone, Pero, Italy) at 37 °C in a 5% CO_2_ incubator. The cell morphology was assessed using an Olympus IX73 microscope (Shinjuku, Tokyo, Japan).

#### 2.2.3. MTT Assay

MTT (3-[4,5-dimethylthiazol-2-yl]-2,5-diphenyltetrazolium bromide) (M5655, Sigma-Aldrich, Saint Louis, MO, USA) was used to evaluate the viability of cells based on their metabolic activity. Approximately 3000 cells per well were seeded into a 96-well plate and treated with biomaterial extracts for 24 h. After exposure, the cell mitochondrial activity was analyzed according to the manufacturer’s instructions. The cells were incubated with MTT solution for 4 h at 37 °C and 5% CO_2_. The medium was removed, and DMSO (Sigma Aldrich, Saint Louis, MO, USA) was added to dissolve the formazan crystals. The absorbance of the samples was measured using a VICTOR Nivo multimode microplate reader (Perkin Elmer, Waltham, MA, USA) at 570 nm wavelength. All the groups were analyzed in eight replicates.

#### 2.2.4. XTT Assay

The XTT test was used to complement the MTT assay to measure the viability of the cells based on their metabolic activity. Approximately 3000 cells per well were seeded into a 96-well plate and treated with biomaterial extracts for 24 h. After exposure, XTT (sodium 3′-[1-(phenylaminocarbonyl)-3,4-tetrazolium]-bis (4-methoxy6-nitro) benzene sulfonic acid hydrate) (11465015001, Roche, Basel, Switzerland) solution and PMS (N-methyl dibenzopyrazine methyl sulfate) (Sigma Aldrich, Saint Louis, MO, USA) electron-coupling reagent were mixed, added into the wells, and incubated for 4 h at 37 °C and 5% CO_2_. The reaction of XTT with mitochondrial dehydrogenases produces a soluble form of formazan, whose absorbance was measured using the multimode microplate reader at 450 nm wavelength. All the groups were analyzed in eight replicates.

#### 2.2.5. Scratch Assay

The cell proliferation under the influence of biomaterial extracts was analyzed by scratch assay (wound healing test). After reaching full confluence, the cells were scratched using a 200 µL pipette tip, then washed with PBS (Corning, New York, NY, USA) to remove non-adherent cells. The scratched area was photographed at 0 h, 6 h, 18 h, and 48 h and was measured using ImageJ software (v. 1.52a). The photographic documentation was prepared microscopically in a phase contrast microscope Olympus IX73 (Shinjuku, Tokyo, Japan). All the groups were analyzed in six replicates.

#### 2.2.6. Flow Cytometry

FxCycle™ PI/RNase Staining Solution (F10797, Thermo Fisher Scientific, Waltham, MA, USA) was used for the flow cytometric analysis of the cell cycle and recognition of the G0/G1, S, and G2/M cell cycle phases. After 24 h of culture with biomaterial extracts, the cells were fixed in 70% ethanol (Sigma-Aldrich, Saint Louis, MO, USA) and incubated with a staining solution for 30 min at room temperature. The samples were analyzed at 488 nm excitation and 585 nm emission wavelengths in three replicates using a Beckman Coulter CytoFLEX cytometer (Indianapolis, IN, USA).

#### 2.2.7. Fluorescence Microscopy

Live and dead cells were visualized on the composite materials using staining performed with fluorescein diacetate (FDA, F1303, Thermo Fisher Scientific, Waltham, MA, USA) and propidium iodide (PI, P3566, Thermo Fisher, Waltham, MA, USA). FDA is converted into fluorescein in live cells, while PI is a fluorescent dye intercalating into nucleic acids and stains only dead cells. After 5 days of culture, the cells were incubated in the dark with the staining solution (FDA and PI in culture medium) at room temperature for 4–5 min. The solution was then removed, and the samples were washed with PBS (Corning, New York, NY, USA) and observed under a fluorescent microscope Olympus IX73 (Olympus, Shinjuku, Tokyo, Japan) equipped with an ORCA-spark Digital CMOS camera (Hamamatsu, Japan).

#### 2.2.8. Scanning Electron Microscopy of Biomaterials Populated by Cells

Before and after preparation of the extracts, PCL, PCL/SHAP, PCL/BIO, and PCL/CH biomaterials were observed with SEM to assess their biodegradation after 24 h at 37 °C. Then, MG-63 were seeded onto the biomaterial samples at a density of 6 × 10^5^ and cultured for 5 days. The biomaterials with and without cells were fixed with 2.5% glutaraldehyde solution in 0.1 M cacodylate buffer at pH 7.2 at room temperature for 1 h. After the fixative was rinsed out, the material was post-fixed in 1% OsO_4_ solution in 0.1 M cacodylate buffer at pH 7.2 for 1 h. The material fixed for SEM was dehydrated in a series of increasing concentrations of alcohol (POCH, Gliwice, Poland) followed by acetone (POCH, Gliwice, Poland). Next, the samples were critical-point dried (Leica EM CPD300, Wetzlar, Germany), then glued to tables and sputtered with gold (Pelco SC-6; Ted Pella Inc., Redding, CA, USA). The observations were made using a Hitachi UHR FE-SEM SU 8010 scanning electron microscope (Hitachi, Tokyo, Japan).

#### 2.2.9. Statistical Analysis

A statistical analysis was performed using Statistica 13.3 (TIBCO, Palo Alto, CA, USA) and GraphPad Prism version 8.0.1 for Windows (GraphPad Software, Boston, MA, USA, www.graphpad.com, accessed on 12 August 2024). The values were expressed as mean and standard deviation for normally distributed variables. Different groups were compared using ANOVA with the Tukey post hoc test. The level of significance was set at 5% for all the statistical tests.

## 3. Results

### 3.1. Biomaterial Analysis

#### 3.1.1. X-ray Diffraction

The XRD analysis showed an amorphous but also crystalline structure of the PCL, hydroxyapatite, bioglass, and chitosan, as well as the resulting composite materials: PCL/SHAP, PCL/BIO, and PCL/CH.

#### 3.1.2. Thermogravimetric Analysis

The thermogravimetric (TG) analysis showed that the highest sample mass loss was shown by PCL (Figure S1A,B), which is related to the thermal degradation of the polymeric material in the test sample. Exothermic transformations were evident, which ultimately led to complete degradation of the sample at temperatures up to 600 °C. For the PCL containing no additives, the weight loss of the sample started at 381 °C. With the introduction of additives such as hydroxyapatite (Figure S1C,D), bioglass (Figure S1G,H), and chitosan (Figure S1K,L), in the amount of 5% relative to the PCL, the largest sample loss was observed for PCL/CH, in which the weight loss started at 248 °C and was highest at 391 °C. For PCL/SHAP, the sample weight loss started at 386 °C, while for the PCL/BIO sample, the weight loss started at 379 °C. The bioglass sample (Figure S1E,F) had the lowest sample mass loss when heated to 600 °C, which is related to its nature as an inorganic material and its high temperature resistance. In the case of chitosan (Figure S1I,J), sample weight loss influenced by its chemical structure was evident but not as significant as for PCL.

#### 3.1.3. BET

Tests carried out on the PCL, PCL/CH, PCL/BIO, and PCL/SHAP samples using a specific surface analyzer showed that the specific surface areas of the obtained composite powders are similar to each other, amounting to 0.46 m^2^/g for PCL, 0.45 m^2^/g for PCL/SHAP, 0.41 m^2^/g for PCL/BIO, and 0.43 m^2^/g for PCL/CH.

#### 3.1.4. Initial Sample Assessment with SEM

**Figure 1:**
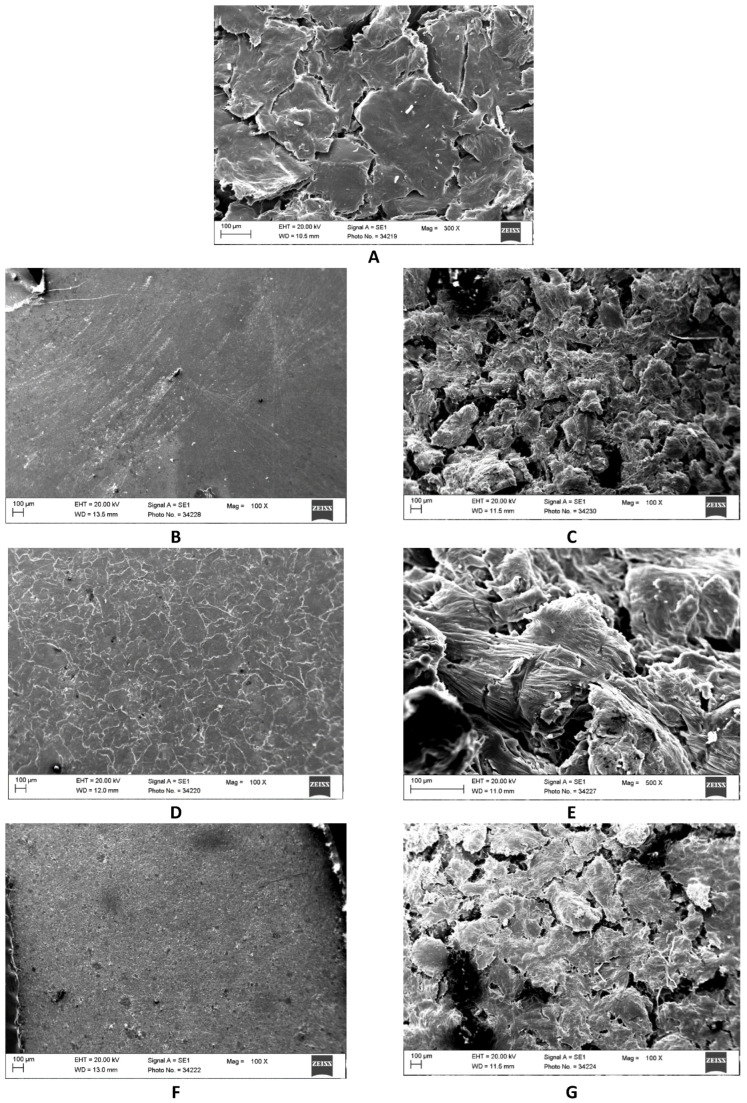
Scanning electron microscopy images of pressed material samples of (**A**) PCL pellets, (**B**) SHAP (hydroxyapatite) powder, (**C**) PCL/SHAP composite, (**D**) BIO (bioglass) powder, (**E**) PCL/BIO composite, (**F**) CH (chitosan) powder, and (**G**) PCL/CH composite. Scale bars = 100 µm.

In scanning electron microscopy, material samples of PCL, PCL/SHAP, PCL/BIO, and PCL/CH showed a characteristic uneven surface, which was related to the initial melting of the polymeric materials, mixing of the polymeric materials with additives, solidification of the materials obtained, subsequent cryogenic grinding of the materials, and finally obtaining compressed shapes (Figure 1). The compaction of powders such as hydroxyapatite, bioglass, and chitosan resulted in the formation of pellets characterized by a significantly fewer surface irregularities compared to composite materials composed of a combination of PCL with hydroxyapatite, bioglass, or chitosan.

#### 3.1.5. Elemental Analysis

The analysis of the chemical composition of the pressed material samples showed that the composite materials, including PCL/SHAP, PCL/BIO, and PCL/CH, are dominated by elements such as oxygen (O) and carbon (C), characteristic of the PCL polymer. The small percentage of elements such as silicon (Si) in the PCL/BIO sample and phosphorus (P) in the PCL/SHAP sample is related to the small 5% share of the additive in relation to PCL (Figure S2).

### 3.2. Impact of Biomaterials on MG-63 Cells

#### 3.2.1. Morphology of Cells

**Figure 2:**
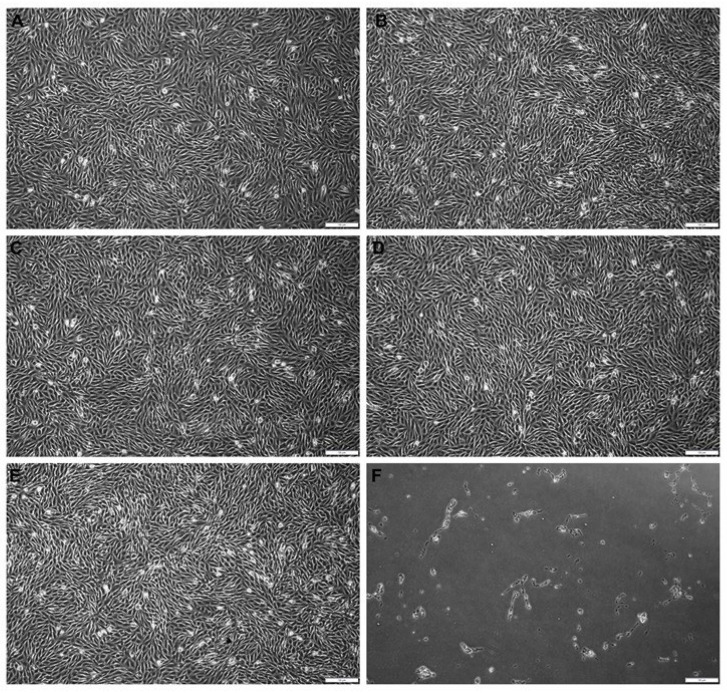
The unchanged morphology of MG-63 cells grown for 24 h in the presence of extracts obtained from biodegradable filament polymers: (**A**) PCL, (**B**) PCL/SHAP, (**C**) PCL/BIO, and (**D**) PCL/CH; (**E**) positive control and (**F**) negative control. Scale bar = 100 µm.

MG-63 cells were examined microscopically under phase contrast to assess the general morphology, vacuolization, detachment, cell lysis, and membrane integrity according to ISO 10993-5:2009(E) [32]. Cells cultured in every type of extract obtained from biodegradable filament polymers had similar fibroblast morphology and remained adherent to the culture dish. The examined extracts did not show any specific reactivity with cells resulting in qualitative morphological changes (Figure 2).

#### 3.2.2. Viability of MG-63 Cells

**Figure 3:**
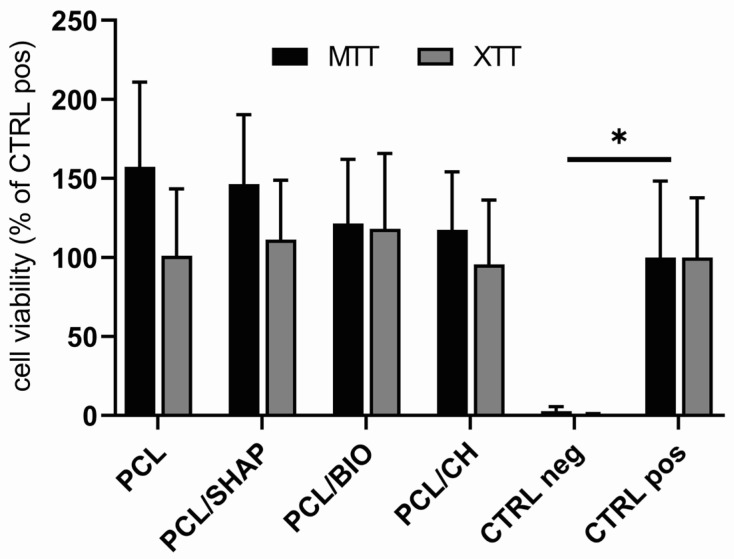
Comparable MG-63 viability based on measurements of mitochondrial activity (MTT and XTT assays) in cells cultured for 24 h in the presence of extracts obtained from biodegradable filament polymers PCL, PCL/SHAP, PCL/BIO, and PCL/CH compared to the positive (CTRL pos) and negative (CTRL neg) controls. The bars represent the means ± standard deviation (SD) (ANOVA with the Tukey post hoc test, * *p* < 0.05 vs. CTRL pos, n = 8).

There were no statistically significant differences in viability assessed by the MTT and XTT assays between the control MG-63 cells and cells cultured for 24 h in the presence of extracts obtained from PCL, PCL/SHAP, PCL/BIO, and PCL/CH biodegradable filament polymers (Figure 3).

#### 3.2.3. Proliferation and Migration of Cells

**Figure 4:**
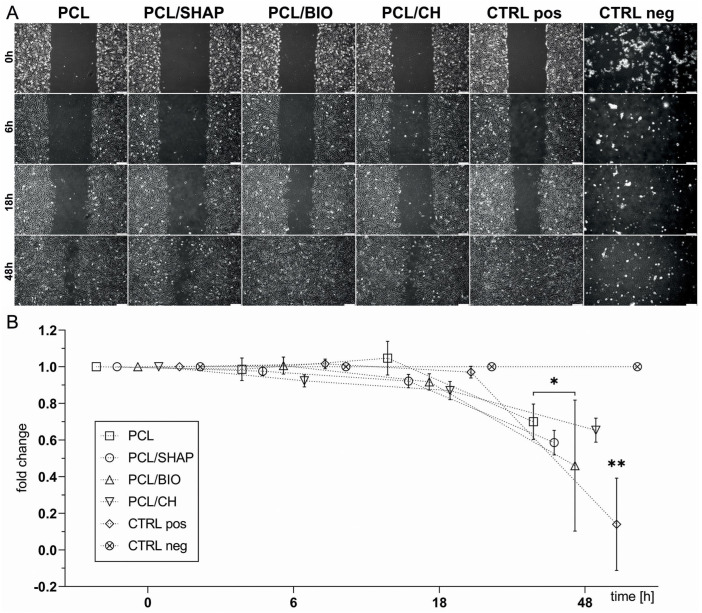
(**A**)**.** Scratch assay of the MG-63 monolayer in response to extracts obtained from biodegradable filament polymers: PCL, PCL/SHAP, PCL/BIO, and PCL/CH compared to the positive and negative controls. Microscopic images representative of each group and time point (0 h, 6 h, 18 h, 48 h); scale bar = 50 µm. (**B**). Scratch area as a multiplicity of the measurement performed at time point 0 h (two-way ANOVA test, * *p* < 0.05; ** *p* < 0.001 for all the other groups; n = 6).

The wound areas in the scratch assay (total number of pixels, n = 6) were calculated as a fold change of the measurements taken at individual time points (6 h, 18 h, and 48 h) as compared to 0 h (Figure 4A). The statistical analysis (two-way ANOVA, *p* < 0.05) showed significant differences between the studied groups. The cells treated with PCL/BIO had a higher proliferation rate than the cells grown in PCL alone (Figure 4B).

#### 3.2.4. Cell Cycle Analysis

**Figure 5:**
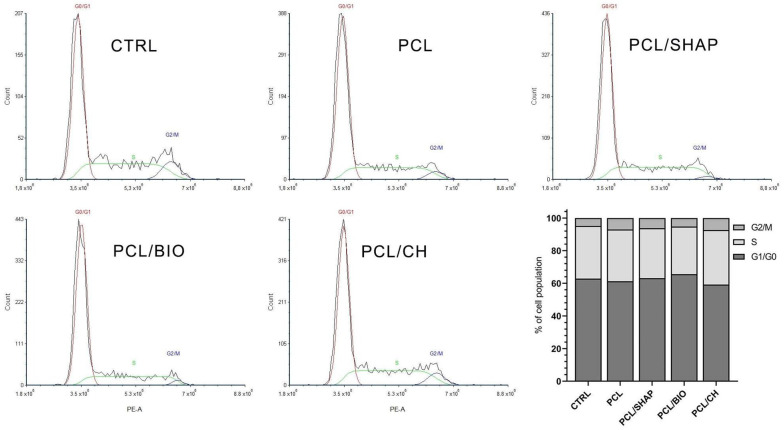
Histograms representative of MG-63 cells in different phases of the cell cycle, cultured for 24 h in the presence of PCL, PCL/SHAP, PCL/BIO, and PCL/CH extracts. The graph shows the mean percentage of cells in different phases of the cell cycle, assessed by flow cytometry (ANOVA, post hoc Tukey, *p* < 0.05, n = 3). CTRL—untreated control.

There were no statistically significant changes between the control and studied groups (ANOVA, post hoc Tukey, *p* < 0.05) in the cell cycle analysis. The cultures incubated in the tested extracts showed a similar percentage of cells representing each phase of the cycle. The highest percentage of cells was observed in the G1/G0 phase (mean 62.4% ± 2.4). In the S and G2/M phases, the mean percentage of cells was 31.5% ± 1.6 and 6.1% ± 1.1, respectively (Figure 5). We did not detect cells with reduced DNA content (sub-G0/G1 cells), specific for apoptotic cells. The analysis showed that the tested extracts did not affect the cell cycle of the tested cells.

#### 3.2.5. Live/Dead Cells Visualized on Composite Materials

**Figure 6:**
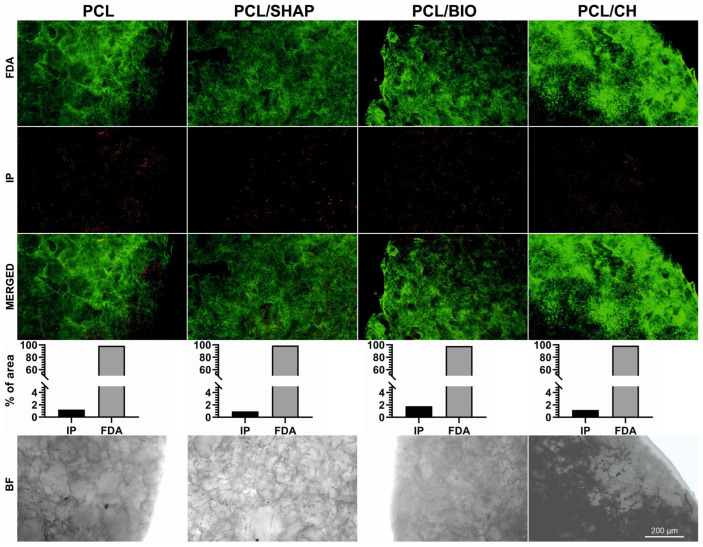
Viability of MG-63 cells grown for 5 days on biodegradable composite materials polymers: PCL, PCL/SHAP, PCL/BIO, and PCL/CH; FDA—fluorescein diacetate (green), IP—propidium iodide (red), BF—bright field; scale bar = 200 µm. The graphs show what percentage of the preparation surface is occupied by dead cells (IP) and live cells (FDA). The proportions between these values were comparable in all the study groups.

Most of the visualized cells on the composite materials were alive, and only some individual dead cells were spotted on each tested material. The result indicates that all the tested biomaterials allowed cells to grow and populate their structure (Figure 6).

#### 3.2.6. Adherence of Cells to Composite Materials

**Figure 7:**
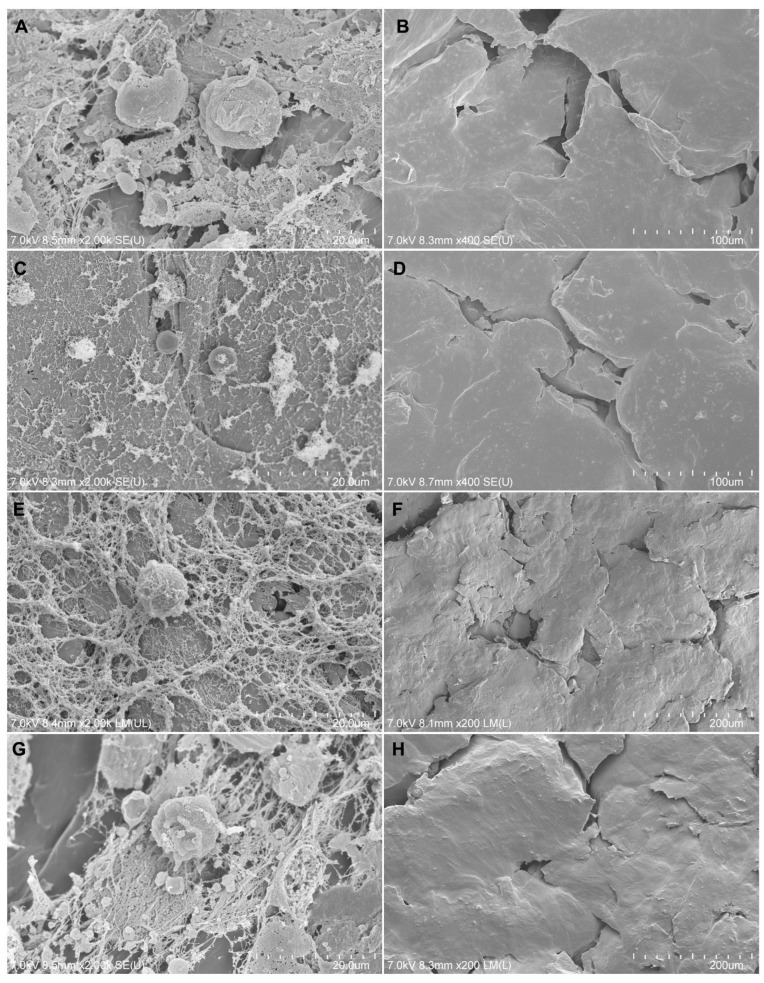
Scanning electron microscopy images of composite materials populated and unpopulated by MG-63 cells. (**A**) PCL with cells, (**B**) PCL without cells, (**C**) PCL/SHAP with cells, (**D**) PCL/SHAP without cells, (**E**) PCL/BIO with cells, (**F**) PCL/BIO without cells, (**G**) PCL/CH with cells, (**H**) PCL/CH without cells. Original scale bars are shown in the lower right corners of the photos.

The morphology of MG-63 cells deposited on the tested composite materials was visualized by SEM (Figure 7). The cells cultured on the polymers had a normal spheroidal shape with characteristic protrusions. It is visible that the cells formed clusters and connections with each other and produced extracellular matrix components on each type of the tested biomaterials. There were no significant differences between the studied groups. MG-63 cells adhered to the tested composite materials in all the groups, confirming that they are not toxic to cells and serve as the basis for stable cell growth. The reference for each group was a composite material sample without cells.

## 4. Discussion

Many approaches have been taken to develop an optimal composite material for the production of bone-specific scaffolds. The requirements for scaffolds used in regenerative medicine specify that the polymers should induce high cell proliferation, promote cell adhesion, and exhibit low pro-apoptotic properties [33]. Polycaprolactone (PCL) has been approved by the U.S. Food and Drug Administration (FDA) and is a widely researched and studied compound in regenerative medicine. When used as a scaffold, its biocompatibility and properties, such as slow biodegradation, allow for successful bone regeneration [34,35]. The main disadvantages of PCL itself are poor mechanical properties, swelling, and possible induction of inflammation in vivo [36,37]. Therefore, new compositions of PCL with some other materials would improve its applicability in bone regeneration.

Some studies have focused on the properties of bioglass, hydroxyapatite [38], or chitosan. It is known that bioactive glass scaffolds have osteoconductive and osteoinductive properties and can be successfully used with autologous adipose or bone marrow stem cells [39,40]. Lee et al. demonstrated the effect of polydopamine-laced hydroxyapatite collagen calcium silicate scaffolds for bone regeneration. They suggested their use especially in critical-sized defects [18]. Tamburaci et al. used polyhedral oligomeric silsesquioxanes (POSS) with chitosan (CS)/Na-carboxymethylcellulose (Na-CMC) to create a scaffold for bone tissue regeneration. Their analysis showed that the scaffold was not cytotoxic [19]. Deng et al. investigated a composition of polylactic–co-glycolic acid/nano-hydroxyapatite (PLGA/nHA) scaffolds with BMP-2 and chitosan (CS). They noticed that PLGA/nHA is characterized by high biocompatibility and a non-toxic effect on cells. Moreover, PLGA/nHA/CS/rhBMP-2 promoted osteogenesis [41]. In this study, we focused on the sensitivity of cells to the potential harmful effects of biomaterials based on PCL and enriched with natural compounds—hydroxyapatite, bioglass, or chitosan—in terms of their usefulness in medical applications. The obtained composites were tested for their potential usefulness in the regeneration and reconstruction of bone tissue.

All the in vitro procedures aimed at assessing the biocompatibility of the developed biomaterials were conducted in accordance with the guidelines of ISO 10993-5:2009(E). As described in the guidelines above, cell–biomaterial interactions should be evaluated by direct and indirect assays [32]. Indirect procedures may include elution tests using extracts from hard materials. Elution tests allow for indirect interaction of the tested material with cells or tissues. By preparing the material extract, we can assess its effects with basic assays to evaluate its cytotoxicity or influence on cell proliferation or morphology.

Our results show that MG-63 cells incubated in PCL/SHAP, PCL/BIO, and PCL/CH composite materials composition extracts did not significantly differ in morphology and cell cycle progression from the cells of the control group and among the groups cultured with different extracts. They efficiently adhered to the surface and showed typical fibroblast morphology. The analysis of the metabolic activity of the cells based on MTT and XTT assays showed that the tested biomaterials did not cause significant changes between the groups. Both tests expressed similar results: MTT, which requires lysis of cells to obtain measurement, and XTT, which does not require cell lysis and gives more sensitive results. Finally, the experiments also showed that the cells successfully adhered to the surfaces of the samples, survived, and retained their morphology. SEM observations revealed a lot of fixed cells on the surfaces of all the material samples, indicating that the surfaces of the tested materials had a porous microstructure, improving cell growth. Furthermore, the proliferation and migration rates have shown some advantages of PCL/BIO over other biomaterials.

In summary, this work addresses the important problem of finding materials that act as tissue substitutes, and its aim was to answer the question whether the introduction of additives such as hydroxyapatite, bioglass, and chitosan can negatively affect the cellular response and thus disqualify them as tissue substitutes. We assume that all the in vitro tested composites are not toxic to the tested cells and are suitable for in vivo evaluation.

It should also be taken into account that, like any in vitro experiment, this study has limitations that can be verified in studies extended to other cell lines derived from other tissues, as well as in preclinical studies in an animal model. In this last context, it does not provide the results of the assessment of the tested biomaterials in the tissues and body fluids of living organisms, including their potential impact during long-term implantation and the possible release of soluble compounds from the biomaterials [42]. In addition to its non-toxicity, animal tests must demonstrate that the biomaterial is non-allergenic and non-pyrogenic. Supporting the observations obtained in in vitro studies with animal model studies will largely allow us to meet the challenges related to the extrapolation of the results of studies carried out on cell lines to in vivo conditions, including the influence of the microenvironment, immune response, mechanical stress, and other natural factors in the surroundings of the transplanted implant.

The final assessment of the investigated biomaterials in terms of their use in bone regeneration requires further studies analyzing the impact of the tested materials on the process of osteogenesis in vivo. The positive result from the biological tests is particularly important for the potential use of these materials in 3D-printing technology (FDM) to create tissue scaffolds, while for SLS printing, they require further optimization in terms of geometry (obtaining spherical structures). Transforming these composite materials into filaments would allow for the printing of tissue scaffolds with defined shapes and no cytotoxic effects on cells. In the long term, significantly beyond the scope of this study, such printed structures could more effectively influence the tissue environment due to the presence of additives like hydroxyapatite, which is known for its osteoconductive properties. However, further research is needed to investigate whether composite materials with the addition of chitosan will exhibit the antibacterial properties for which chitosan is known. This aspect should also be verified in future studies.

## 5. Conclusions

Composite materials made at high temperatures from polycaprolactone with additions of hydroxyapatite, bioglass, or chitosan, just like polycaprolactone itself, are neutral to cells and promote their growth and proliferation. All the investigated materials could be used for scaffold production and warrant further investigation in vivo, but PCL with bioglass additives might be the best option.

## Data Availability

Data are contained within the article.

## Supplementary Materials

The following supporting information can be downloaded at: https://www.mdpi.com/article/10.3390/biomedicines12091949/s1, Figure S1: XRD analysis of biomaterials. Figures show XRD and DTA/TG plots for: PCL (**A**,**B**); PLC composite with hydroxyapatite (**C**,**D**); bioglass (**E**,**F**); PCL composite with bioglass (**G**,**H**); chitosan (**I**,**J**); PCL composite with chitosan (**K**,**L**); Figure S2. Elemental analysis. Analysis of chemical composition (EDS) of the pressed samples of: (**A**) PCL polymer, (**B**) hydroxyapatite powder (SHAP), (**C**) PCL/SHAP composite material pellet. (**D**) bioglass powder, (**E**) PCL/BIO composite material pellet, (**F**) chitosan polymer, (**G**) PCL/CH composite material pellet; Table S1: Sample sterilization conditions.
