# Supplementary material for: Toxicological Assessment of Biodegradable Poli-ε-Caprolactone Polymer Composite Materials Containing Hydroxyapatite, Bioglass, and Chitosan as Potential Biomaterials for Bone Regeneration Scaffolds
Source: Biomedicines. 2024 Aug 26;12(9):1949. doi: 10.3390/biomedicines12091949 (PMC11428512; doi:10.3390/biomedicines12091949)
Supplement: Supplementary file 1 [file biomedicines-12-01949-s001.zip › biomedicines-3137813-supplementary.pdf]

biomedicines

MDPI

Supplementary materials:

Toxicological Assessment of Biodegradable Poli-ε-caprolactone Polymer Composite Materials Containing Hydroxyapatite, Bioglass and Chitosan as Potential Biomaterials for Bone Regeneration Scaffolds

Aleksandra Skubis-Sikora[1†], Andrzej Hudecki[2†], Bartosz Sikora[1], Patrycja Wieczorek[1], Mateusz Hermyt[1], Marek Hreczka[2], Wirginia Likus[3], Jarosław Markowski[4], Krzysztof Siemianowicz[5], Aleksandra Kolano-Burian[2], Piotr Czekaj[1*]

[1] Department of Cytophysiology, Chair of Histology and Embryology, Faculty of Medical Sciences in Katowice, Medical University of Silesia in Katowice, 40-055 Katowice, Poland; askubis@sum.edu.pl (A.S.-S.); bsikora@sum.edu.pl (B.S.); pszmytkowska@sum.edu.pl (P.W.); mhermyt@sum.edu.pl (M.H.)
[2] Łukasiewicz Research Network-Institute of Non-Ferrous Metals, 44-121 Gliwice, Poland; andrzej.hudecki@imn.lukasiewicz.gov.pl (A.H.); marek.hreczka@imn.lukasiewicz.gov.pl (M.H.); aleksandra.kolano-burian@imn.lukasiewicz.gov.pl (A.K.-B.)
[3] Department of Anatomy, Faculty of Health Sciences in Katowice, Medical University of Silesia in Katowice, 40-055 Katowice, Poland; wirginia.likus@gmail.com
[4] Department of Laryngology, Faculty of Medical Sciences in Katowice, Medical University of Silesia in Katowice, 40-055 Katowice, Poland; jmarkowski@sum.edu.pl
[5] Department of Biochemistry, Faculty of Medical Sciences in Katowice, Medical University of Silesia in Katowice, 40-055 Katowice, Poland; ksiemian@gmail.com
* Correspondence: pcz@sum.edu.pl
† These authors contributed equally to this work.

Table S1. Sample sterilization conditions.

No.	Name Value	No.
1	Prescribed dose	15 kGy
2	Transporter	0,747 m/min
3	Prescribed current	600 mA
4	Energy	10 MeV
5	Calibration factor	11,2
6	Sampling	0,3s
7	Control	AUTO
8	Average dose	15 kGY +/- 0,613 %
9	Average transporter	0,758 m/min +/- 1,23
10	Average current	609 mA +/- 1,15 %

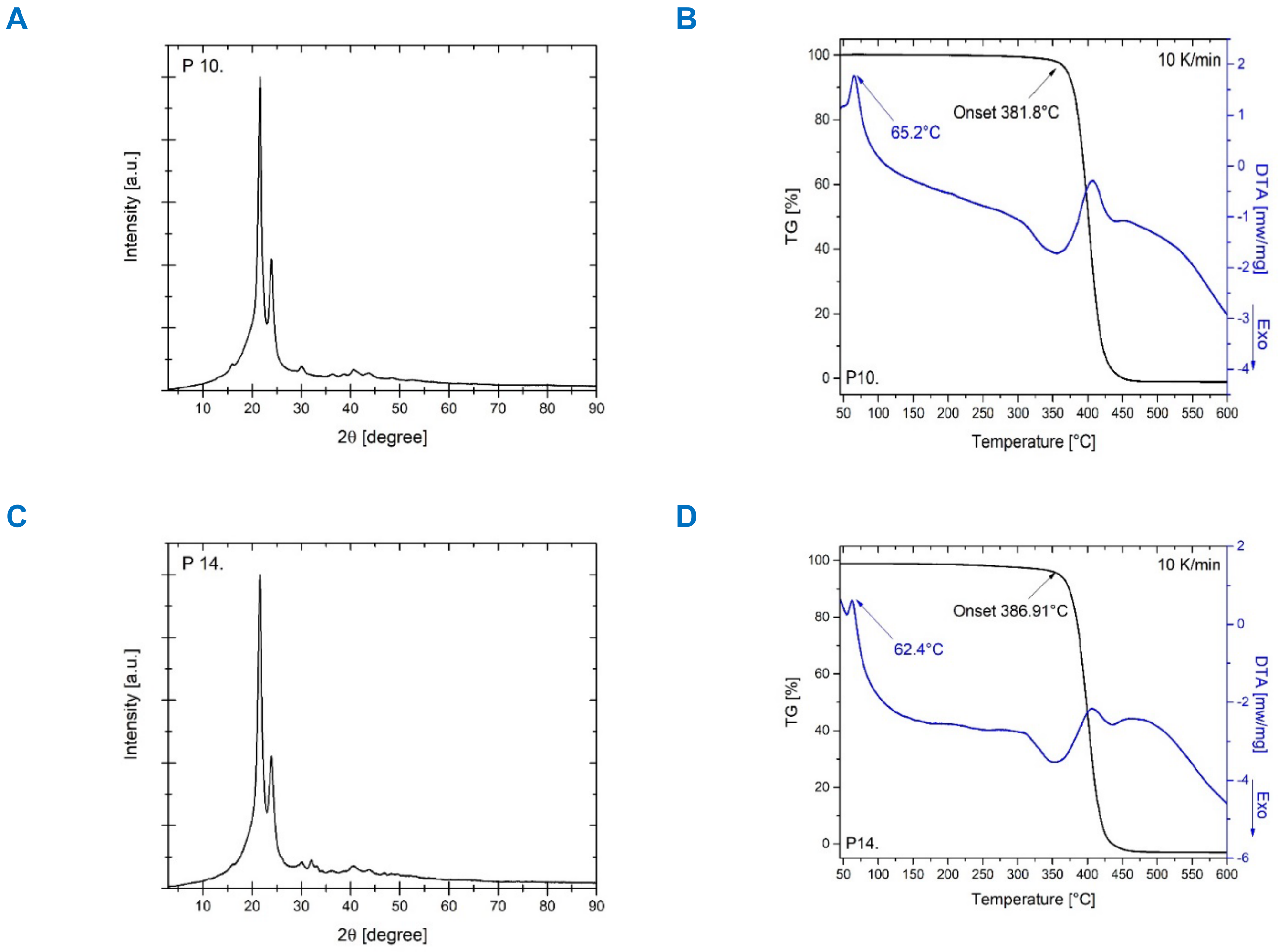

E

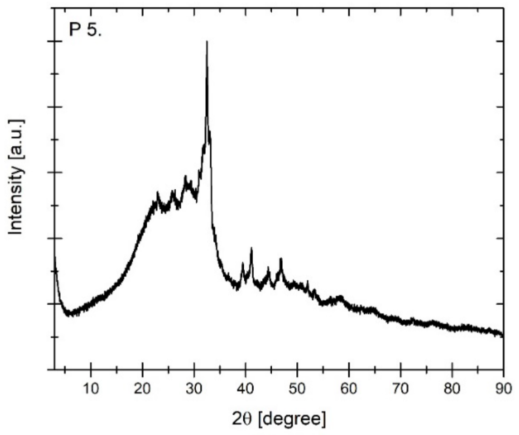
P 5.
Intensity [a.u.]
2θ [degree]
10 20 30 40 50 60 70 80 90

F

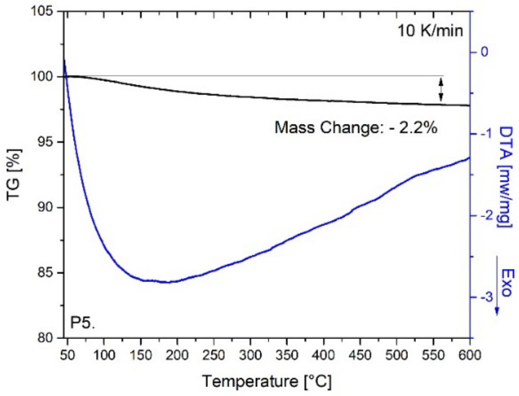
10 K/min
Mass Change: - 2.2%
P5.
TG [%]
DTA [mw/mg]
Exo
105 100 95 90 85 80
0 -1 -2 -3
50 100 150 200 250 300 350 400 450 500 550 600
Temperature [°C]

G

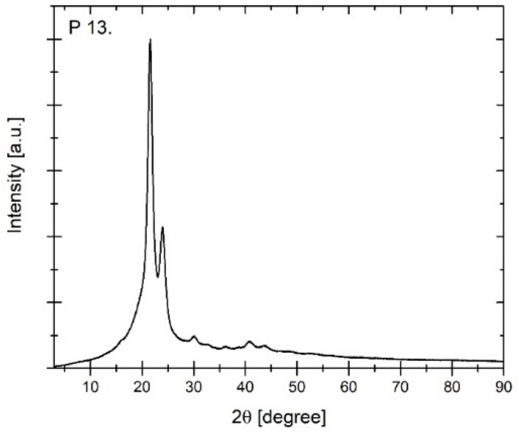
P 13.
Intensity [a.u.]
2θ [degree]
10 20 30 40 50 60 70 80 90

H

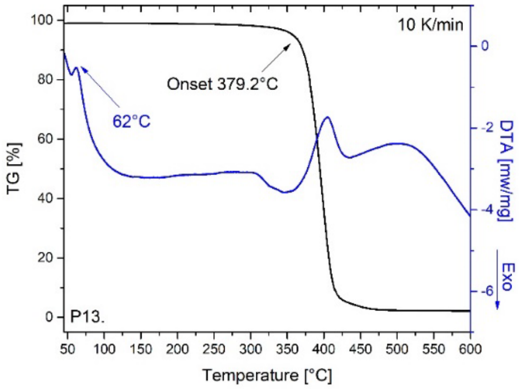
10 K/min
Onset 379.2°C
62°C
P13.
TG [%]
DTA [mw/mg]
Exo
100 80 60 40 20 0
0 -2 -4 -6
50 100 150 200 250 300 350 400 450 500 550 600
Temperature [°C]

I

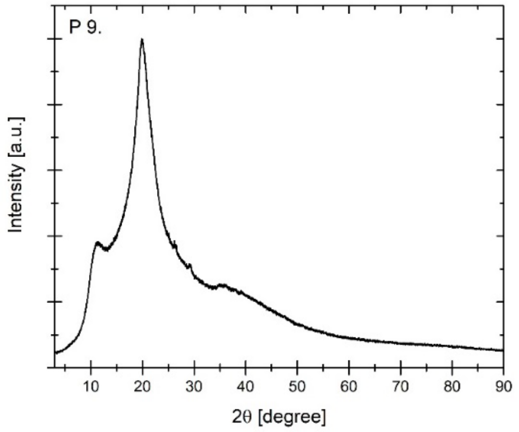
P 9.
Intensity [a.u.]
2θ [degree]
10 20 30 40 50 60 70 80 90

J

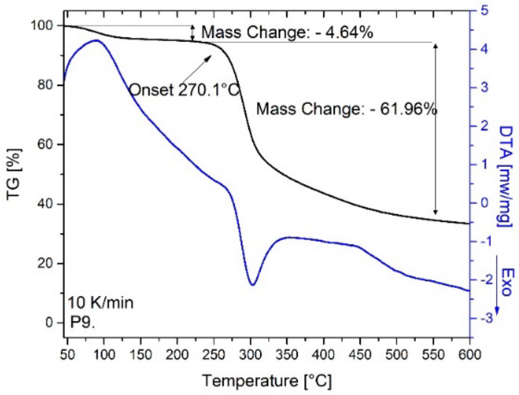
Mass Change: - 4.64%
Onset 270.1°C
Mass Change: - 61.96%
10 K/min
P9.
TG [%]
DTA [mw/mg]
Exo
100 80 60 40 20 0
5 4 3 2 1 0 -1 -2 -3
50 100 150 200 250 300 350 400 450 500 550 600
Temperature [°C]

K

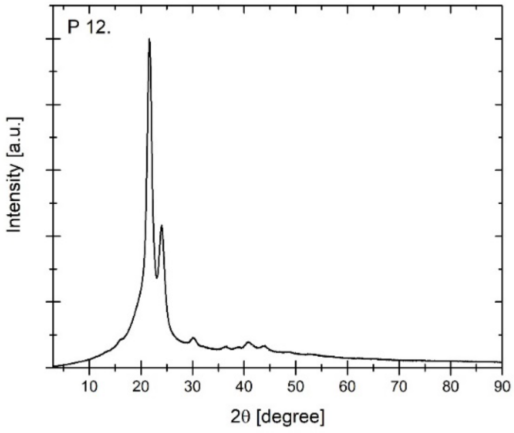

L

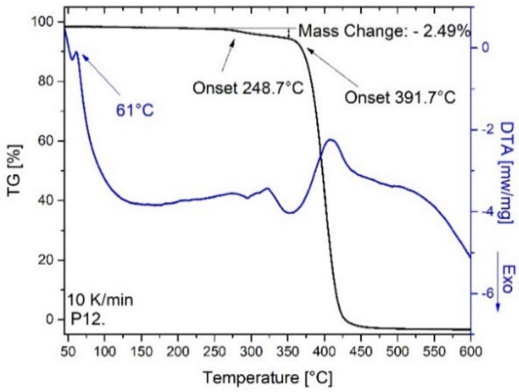

Figure S1. XRD analysis of biomaterials. Figures show XRD and DTA/TG plots for: PCL (A, B); PLC composite with hydroxyapatite (C, D); bioglass (E, F); PCL composite with bioglass (G, H); chitosan (I, J); PCL composite with chitosan (K, L).

A

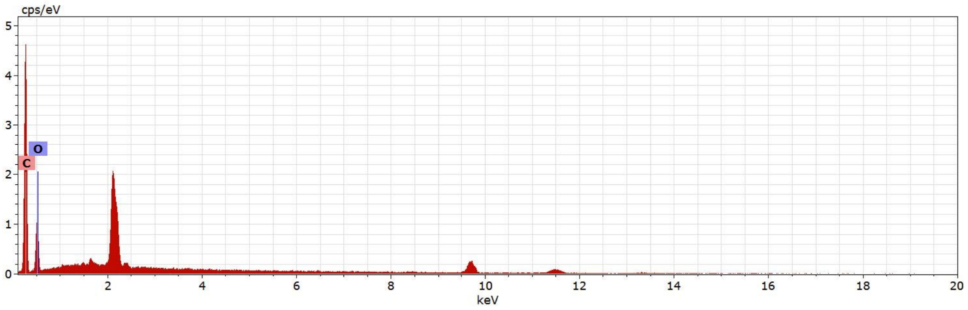

Element	series	[wt.%]	[norm. wt.%]	[norm. at.%]	Error in wt.% (3 Sigma)
**Carbon**	K-series	63,46311	63,46311	69,82261	25,31501
**Oxygen**	K-series	36,53689	36,53689	30,17739	17,91969
	Sum:	100	100	100	

B

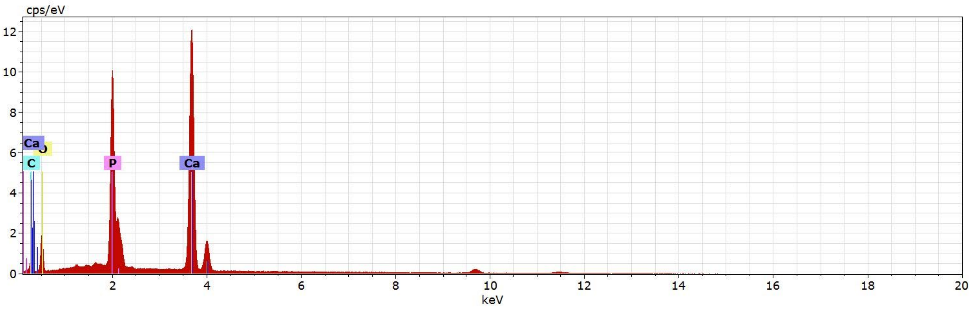

Element	series	[wt.%]	[norm. wt.%]	[norm. at.%]	Error in wt.% (3 Sigma)

**Carbon**	K-series	3,605107	5,862055	11,7026	2,16034
**Oxygen**	K-series	19,70102	32,03469	48,00965	8,238406
**Calcium**	K-series	27,23803	44,29018	26,49799	2,467559
**Phosphorus**	K-series	10,95487	17,81308	13,78976	1,354673
	Sum:	61,49903	100	100	

C

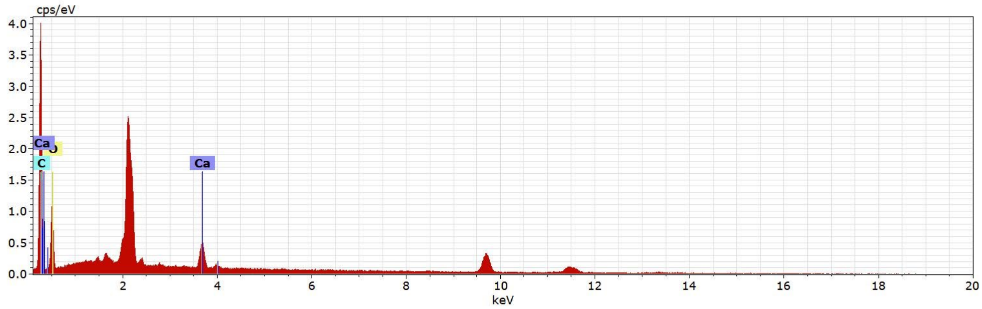

**Element**	**series**	**[wt.%]**	**[norm. wt.%]**	**[norm. at.%]**	**Error in wt.% (3 Sigma)**
**Carbon**	K-series	57,80918	57,80918	65,7516	22,49445
**Oxygen**	K-series	38,72775	38,72775	33,06795	17,63979
**Calcium**	K-series	3,463074	3,463074	1,180442	0,432228
	Sum:	100	100	100	

D

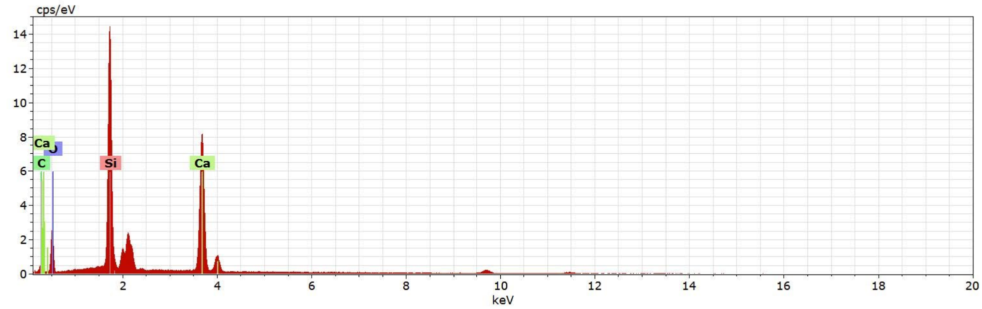

**Element**	**series**	**[wt.%]**	**[norm. wt.%]**	**[norm. at.%]**	**Error in wt.% (3 Sigma)**
**Carbon**	K-series	3,809976	7,050505	13,00162	2,661629
**Oxygen**	K-series	19,62222	36,31167	50,26886	8,65794
**Calcium**	K-series	18,17501	33,63354	18,58759	1,689358
**Silicon**	K-series	12,43114	23,00429	18,14192	1,67711
	Sum:	54,03834	100	100	

E

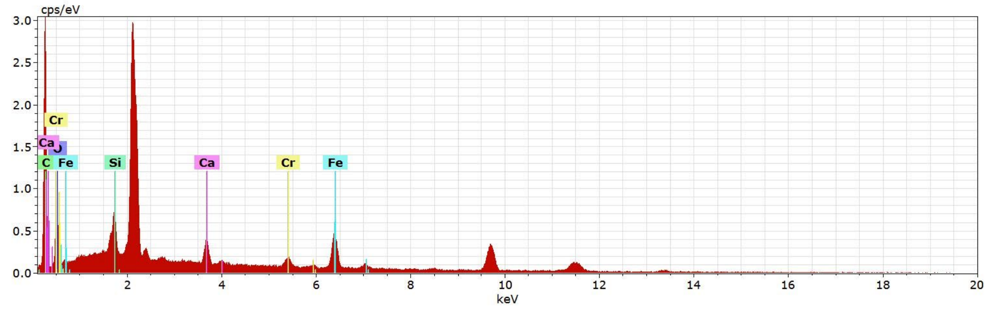

Element	series	[wt.%]	[norm. wt.%]	[norm. at.%]	Error in wt.% (3 Sigma)
**Carbon**	K-series	13,45993	57,19253	71,24602	5,476473
**Oxygen**	K-series	5,841176	24,81971	23,21098	2,919882
**Iron**	K-series	2,85901	12,1482	3,254713	0,333104
**Calcium**	K-series	0,499982	2,124472	0,793132	0,130367
**Chromium**	K-series	0,46499	1,975788	0,568553	0,129028
**Silicon**	K-series	0,409333	1,739296	0,926599	0,134679
	Sum:	23,53442	100	100	

F

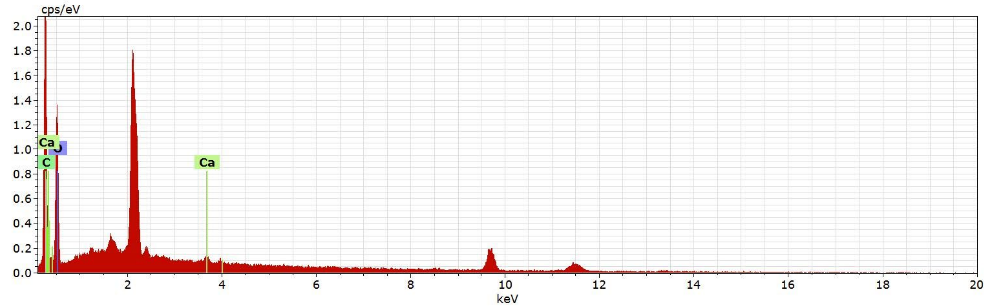

Element	series	[wt.%]	[norm. wt.%]	[norm. at.%]	Error in wt.% (3 Sigma)
**Carbon**	K-series	49,54058	49,54058	56,80067	20,79177
**Oxygen**	K-series	50,00943	50,00943	43,04471	22,3275
**Calcium**	K-series	0,449986	0,449986	0,15462	0,161412
	Sum:	100	100	100	

G

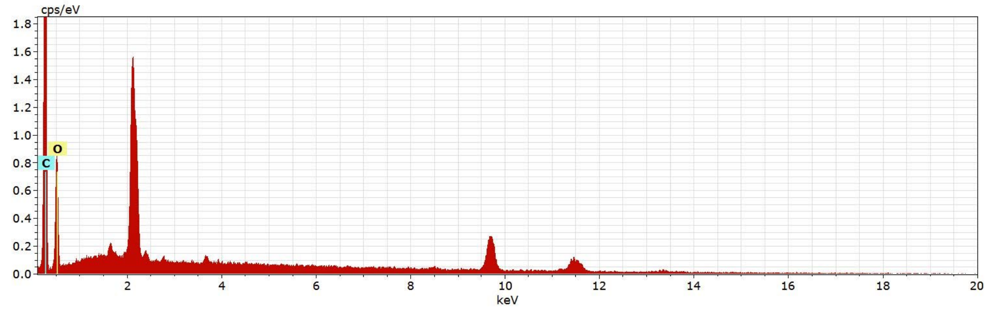

Element	series	[wt.%]	[norm. wt.%]	[norm. at.%]	Error in wt.% (3 Sigma)
**Carbon**	K-series	63,23227	63,23227	69,61271	24,85136
**Oxygen**	K-series	36,76773	36,76773	30,38729	17,56936
	Sum:	100	100	100	

Figure S2. Elemental analysis. Analysis of chemical composition (EDS) of the pressed samples of: A - PCL polymer, B – hydroxyapatite powder (SHAP), C – PCL/SHAP composite material pellet. D – bioglass powder, E – PCL/BIO composite material pellet, F – chitosan polymer, G – PCL/CH composite material pellet.
